# Effectiveness of Omega-3 Supplement on Lipid Profile and Lipid Peroxidation in Kidney Allograft Recipients

**DOI:** 10.5812/numonthly.9384

**Published:** 2013-06-20

**Authors:** Hamid Tayebi Khosroshahi, Seyed Ehsan Mousavi Toomatari, Sara Akhavan Salamat, Giti Davar Moin, Sattar Najafi Khosroshahi

**Affiliations:** 1 Chronic Kidney Disease Research Center, Imam Reza Hospital, Tabriz University of Medical Sciences, Tabriz, IR Iran; 2 Students’ Research Committee, Tabriz University of Medical Sciences, Tabriz, IR Iran; 3 Zahravi Pharmaceutical Company, Tabriz, IR Iran

**Keywords:** Fatty Acids, Omega-3, Lipid Peroxidation, Kidney Transplantation

## Abstract

**Background:**

Omega-3 fatty acids carry major roles in mediating inflammation, immune response, lipid peroxidation and lipoprotein metabolism. Diversity of health benefits have been attributed to dietary supplementation with omega-3 fatty acids in transplant and nontransplant settings. Several studies in renal transplantation have suggested that supplementation with omega-3 fatty acids may lead to significant clinical benefits. However, the extents of these benefits are variable and published data had not coincided with positive findings.

**Objectives:**

The aim of this study was to evaluate the effectiveness of omega-3 supplementation on the lipid profile and lipid peroxidation in patients underwent kidney transplant.

**Patients and Methods:**

Thirty cases had been selected with stable allograft function following at least six months of transplantation. The serum levels of lipids including triglyceride, low density lipoprotein (LDL), high density lipoprotein (HDL), very low density lipoprotein (VLDL), total cholesterol and indices of lipid peroxidation (malondialdehyde and APO a1) were measured by biochemical techniques at the baseline. Two months following prescription of oral omega-3 (3 g/day), the biochemical measurements were repeated and the differences were analyzed.

**Results:**

Of thirty patients, 12 were male and 18 were female with the mean age of 45.3 ± 13.0 (18-65) years. At the baseline, the serum levels of MDA and APO B were 3.5 ± 1.3 and 148.3 ± 20.4 ng/dL respectively. At the end of two months following intervention, they were 3.2 ± 1.2 and 145.7 ± 19.0 ng/dL, respectively (P > 0.05). Correspondingly, at the baseline the serum levels of triglyceride, LDL, VLDL and total cholesterol were 171.1 ± 58.7, 106.9 ± 31.8, 42.2 ± 4.0, 145.7 ± 33.2 and 181.2 ± 35.1 mg/dL and after intervention they were 162.4 ± 82.5, 99.4 ± 35.1, 44.6 ± 6.3, 140.3 ± 33.1 and 170.9 ± 38.3 mg/dL, respectively (P > 0.05). There was no significant difference between the males and females in this instance.

**Conclusions:**

Our results seem to indicate that oral omega-3 may promote the lipid profile and indices of lipid peroxidation in patients following kidney transplantation however extents of these effects are not significant.

## 1. Background

One of the common metabolic disorders following kidney transplantation is hyperlipidemia and lipid profile disorder and it is associated with poor prognosis (1). In kidney transplanted patients, cardiovascular deaths account for the majority of mortalities (2). Some causes of death include increased cardiovascular risk such as calcium and phosphor metabolic failure, chronic increase in fluid volume, hyper hemocysteinemia and permanent oxidative stresses (2). An increased level of lipid peroxidation occurs in ESRD patients who lead to atherosclerosis (3, 4). Furthermore, hyperlipidemia may increase incidence of rejection following kidney transplantation via oxidative stresses (5). Omega-3 is an unsaturated fatty acid with two or three strains that the human body cannot produce and it is found in fish oil (6, 7). Hyperlipidemia occurs less often in people who consume fish. Correspondingly, their level of omega-3 and HDL are higher than others and level of LDL is lower (8). The renal transplanted patients may benefit from dietary fish oil due to a decrease in TNF-alpha, IL-1 and IL-2 (9). Omega-3 is thought to have antioxidant properties through its effect on oxide LDL particles, but overall, this has not been established as a certainty (3, 4). Probably omega-3’s anti-thrombotic and anti-inflammatory effect is caused by inflammatory factors such as IL-1B and IL-2 reduction. Also, omega-3 change the lipo-oxygenase and cyclo-oxygenase pathway which leads to leucocytes’ function changes and affects inflammatory factors (10). Omega-3‘s role was studied in renal failure and kidney transplanted patients and different results were obtained (11).

## 2. Objectives

The aim of this study was to assess effect of omega-3 on lipid profile in kidney transplanted patients. If there is a positive effect of omega-3 on serum lipids, unwanted complications can be prevented in these patients with a prophylactic prescription.

## 3. Patients and Methods

In this study, 30 kidney allograft recipients who had the necessary inclusion criteria's were selected. Omega-3 (Zahravi pharmaceutical company, Tabriz, Iran) as 1 gram soft gel capsule was administered three grams per day after meals for two months. This study was performed from February 2009 to February 2010 in university hospitals of Tabriz University of Medical Sciences. Inclusion criteria were defined as 1) Individual consent to participate in this study; 2) Adult kidney allograft recipients; 3) No history of treatment with omega-3 or consumption of fish; 4) A period of at least 6 months after kidney transplantation. We excluded patients who had drug intolerance, active infectious or inflammatory disease. These patients were on maintenance immunosuppressive therapy including Cyclosporine (Imminural, Zahravi pharmaceutical company, Tabriz, Iran) Mycophenolate mofetile (F. Hoffmann-La Roche Ltd, as well as Basel, Switzerland) and Prednisolone. Serum level of malonyl dialdehyde (MDA), apo protein A1 (APO A1), triglyceride (TG), low density lipoprotein (LDL), high density lipoprotein (HDL), very low density lipoprotein (VLDL) and total cholesterol, Na+, K+, serum creatinine (Cr) and blood urea nitrogen (BUN) were measured at baseline and following two months at the end of the study. Lipid profile and lipid peroxidation status and other laboratory parameters were measured by standard biochemical methods. Malonyl dialdehyde was assessed as malonyl dialdehyde like material (MDA-LM) by the thiobarbituric acid assay, according to the method which was defined by Smith et al. (2). Briefly, 200 UL of plasma was treated with an equal volume of perchloric acid and heated for five minutes at 100°C to precipitate the proteins. After centrifugation at 3000 g for 10 minutes, 300 UL of sample supernatant was added to 300 UL of thiobarbituric acid and perchloric acid aqueous solution (0.66% and 1P15% v/v respectively). The color reaction was achieved by heating for 15 minutes at 100°C. A blank was made for each plasma specimen under the same conditions, except that thiobarbituric acid was not added to the reaction medium. Absorbance was measured at 532 nm and corrected for the absorbance of the blank, and the MDA-LM was determined by reference to a standard curve.

Apo B was measured based on the reaction of a sample containing human Apo B and a specific antiserum to form an insoluble complex which can be measured turbidimetrically at 340 nm. By constructing a standard curve from the absorbance of standards, the concentration of apo B in the samples can be determined. Changes in serum levels of parameters related to lipid profile and lipid peroxidation was compared in both sexes. The study protocol was approved by the institutional ethic committee.

### 3.1. Statistical Analysis

Continuous data with normal distribution are given as mean ± standard deviation, otherwise as median. Student paired t test was used for comparing the significance change of data before and after treatment and Chi-square or Fisher exact test for testing the significance of percentages. A P value of 0.05 or less was considered significant.

## 4. Results

In the present survey, 30 kidney allograft recipients with stable allograft function who qualified based on the inclusion criteria were enrolled the study. Out of 30 patients, 12 were male and 18 female with the mean age of 45.3 ± 13 (18-65). Mean serum MDA was 3.5 ± 1.3 ng/mL before omega 3 administration and reached 3.2 ± 1.2 ng/mL following two months of omega 3 supplementations that, at the end of study, showed no significant change (P > 0.05). Serum Apo B level was 148.3 ± 20.4 ng/mL before the study and 145.7 ± 19 ng/mL at the end of study without significant changes (P > 0.05). There was a non-significant change in lipids level, serum creatinine (Cr) and blood urea nitrogen (BUN) and other laboratory results at the end of the study (P > 0.05) (Table 1). There was also no significant change in hemoglobin (Hb), hematocrit (Hct), leukocyte and platelet count during the study. Coagulative tests and serum electrolyte were also not significantly changed. Lipid profile parameters were measured before and after intervention in both genders but there were no significant differences between male and female in this respect (P > 0.05) (Table 2). There was no acute rejection episode or deterioration of allograft function in our cases during the study.

**Table 1.** Lipids Level and Renal Function Tests Before and After two Months Omega 3 Supplementation

Variable	Baseline	After Intervention	Differences	P value
**TG** **^a^**	171.1 ± 58.7 mg/dL	162.4 ± 82.5 mg/dL	8.7 ± 49.0 mg/dL	NS ^a^
**LDL** **^a^**	106.9 ± 31.8 mg/dL	99.4 ± 35.1 mg/dL	7.9 ± 41.8 mg/dL	NS
**VLDL** **^a^**	145.7 ± 33.2 mg/dL	140.3 ± 33.1 mg/dL	5.4 ± 21.3 mg/dL	NS
**HDL ** **^a^**	42.2 ± 4.0 mg/dL	44.6 ± 6.3 mg/dL	-2.2 ± 6.0 mg/dL	NS
**TC** **^a^**	181.2 ± 35.1 mg/dL	170.9 ± 38.3 mg/dL	10.4 ± 37.4 mg/dL	NS
**BUN** **^a^**	22.5 ± 6.5 mg/dL	21.3 ± 6.2 mg/dL	0.7 ± 5.7 mg/dL	NS
**Cr** **^a^**	1.5 ± 0.3 mg/dL	1.5 ± 0.3 mg/dL	0.0 ± 0.2 mg/dL	NS
**Na** **^a^**	137.7 ± 2.1 meq/dL	138.3 ± 2.6 meq/dL	-0.6 ± 3.1 meq/dL	NS
**K** **^a^**	4.6 ± 0.3 meq/dL	4.6 ± 0.2 meq/dL	0.1 ± 0.3 meq/dL	NS

^a^Abbreviations: BUN, blood urea nitrogen; Cr, creatinine; HDL, high density lipoprotein; K, potassium; LDL, low density lipoprotein; Na, sodium; NS, not significant; VLDL, very low density lipoprotein; TG, triglyceride

**Table 2.** Comparison of Lipid Profile Parameters Before and After Intervention in Male and Female

Parameters Changes	Gender		P value
	Male	Female	
**MDA** **^a^**	0.1 ± 1.3 ng/dL	3.5 ± 1.3 ng/dL	NS (0.602)
**APO ** **^a^**	1.8 ± 8.3 ng/dL	3.5 ± 1.3 ng/dL	NS (0.755)
**TG** **^a^**	8.5 ± 36.5 mg/dL	3.5 ± 1.3 mg/dL	NS (0.917)
**LDL ** **^a^**	6.9 ± 30.0 mg/dL	3.5 ± 1.3 mg/dL	NS (0.586)
**HDL ** **^a^**	-2.7 ± 4.5 mg/dL	3.5 ± 1.3 mg/dL	NS (0.263)
**VLDL ** **^a^**	7.4 ± 6.8 mg/dL	3.5 ± 1.3 mg/dL	NS (0.884)
**TC ** **^a^**	-2.7 ± 19.4 mg/dL	3.5 ± 1.3 mg/dL	NS (0.158)

^a^Abbreviations: Apo, apo protein; HDL, high density lipoprotein; LDL, low density lipoprotein; MDA, malonyl dialdehyde; NS, not significant; VLDL, very low density lipoprotein; TG, triglyceride

## 5. Discussion

Chronic kidney disease (CKD) is one of the most important public health problems which leads to dialysis and transplantation (12). The incidence of cardiac death in dialysis and transplant patients is higher than the general population which can be attributed to hyperlipidemia, hypertension, diabetes mellitus, smoking and other reasons (13). One of the common problems in patients before and after kidney transplantation are lipid abnormalities which can lead to development and progression of CVR and chronic graft dysfunction (14). Oxidative stress causes the most pathological changes in the kidney, initially followed by an inflammatory reaction (12). Some studies demonstrated the effect of omega-3 on dyslipidemia, lipid and protein peroxidation, and antioxidant and anti-inflammatory defense in patients with chronic renal failure (CRF) (15-18). Fish oil is rich in omega-3, polyunsaturated fatty acids (PUFAs), which has a therapeutic role in the treatment of hypertriglyceridemia (11, 19) and a favorable effect on lipoprotein profile in patients with CRF (20). However, other studies demonstrated omega-3 supplementation has no benefit on graft survival among patients who had kidney transplants. No reduction in either early or late acute rejections was found with fish oil supplementation (21).

In this study, we assessed lipid profile and peroxidation changes in kidney transplantation, 2 months following omega-3 prescriptions with doses of 3 g/day. Although, several studies were done regarding the effect of omega-3 (fish oil) in kidney transplanted patients, but there was no study regarding parameters related to lipid peroxidation change after omega-3 intake. Lim and et al in a meta-analysis assessed the results of 16 studies. Their findings showed that omega-3 prescriptions in kidney allograft recipients did not have a significant effect on organ or patients’ survival, acute rejection rate or kidney’s function. When compared with a placebo, omega-3 was associated with a lower diastolic blood pressure and higher HDL level. Therefore there was no sufficient evidence from this study to recommend omega-3 intake to improve renal function (11). Our results are compatible with this study, i.e., there was not any change in allograft function following omega-3 ingestion. In other study, Hernandez and et al. studied dietary fish oil effects on acute rejection rate in two groups (fish oil group and control group) during first 3 months after transplantation. There is no significant difference between two groups in terms of acute rejection rate, graft survival and renal function (9). There was no acute rejection episode in our cases during the study. Nevertheless, serum triglyceride level reduction in the case group was insignificantly higher than in the control group (9). The results of our study are similar to the above, i.e., that there was insignificant serum triglyceride level reduction. Cortinovis and et al. in a study demonstrated that omega-3 prescription did not have influence on serum triglyceride and total cholesterol level and did not recommend its prescription (22). In other systematic reviews and meta-analysis, Tatsioni and et al. assessed 16 studies regarding the effect of fish oil supplementation on kidney transplantation. This study demonstrated that omega-3 consumption had little effect on reducing serum triglyceride levels. As well, its effect on other parameters such as total cholesterol, HDL and LDL were not significant (23).

In a study by Kooijmans et al. 25 kidney transplanted patients that ingest cyclosporine were assessed. Omega-3 supplementation was prescribed with dose of 6 g/day in these patients and followed up for 12 months. This study demonstrated that there were no significant differences between two groups (case and control groups) regarding kidney function parameters, blood pressure and acute rejection and laboratory parameters. For that reason, they recommended that omega-3 should not be used for kidney transplanted patients (24). Our study indicates better outcomes after the administration of omega-3, but there were no significant differences between two measurements (baseline and 2 months following intervention). This study encountered few limitations. The patient population was small which may affect the results. The duration of the study was short when compared to other studies in this area. Moreover, Cyclosporine levels were not measured in our investigation.

In conclusion, consistent with previous studies, this study didn’t show any significant effect of omega 3 supplementation on lipids profile and lipid peroxidation. Further studies with more sample size and a longer duration follow up is recommended.
